# Application of miRNA Biomarkers in Predicting Overall Survival Outcomes for Lung Adenocarcinoma

**DOI:** 10.1155/2022/5249576

**Published:** 2022-09-12

**Authors:** Tingting Li, Huanqing Liu, Chunsheng Dong, Jun Lyu

**Affiliations:** ^1^Department of Pharmacy, Xi'an Chest Hospital, Xi'an, Shaanxi, China; ^2^Northwestern Polytechnical University, Xi'an, Shaanxi, China; ^3^School of Computer Science, Shaanxi Normal University, Xi'an, Shaanxi, China; ^4^Department of Clinical Research, The First Affiliated Hospital of Jinan University, Guangzhou, Guangdong, China

## Abstract

**Background:**

With the development of research, the importance of microRNAs (miRNAs) in the occurrence, metastasis, and prognosis of lung adenocarcinoma (LUAD) has attracted extensive attention. This study is aimed at predicting overall survival (OS) results through bioinformatics to identify novel miRNA biomarkers and hub genes.

**Materials and Methods:**

The data of LUAD-related miRNA and mRNA samples was downloaded from The Cancer Genome Atlas (TCGA) database. Upon screening and pretreatment of initial data, TCGA data were analyzed using R platform and a series of analytical tools to identify biomarkers with high specificity and sensitivity.

**Results:**

7 miRNAs and 13 hub genes that had strong relation to the overall surviving status were identified in patients with LUAD. The expression of seven miRNAs (hsa-miR-19a-3p, hsa-miR-126-5p, hsa-miR-556-3p, hsa-miR-671-5p, hsa-miR-937-3p, hsa-miR-4664-3p, and hsa-miR-4746-5p) could apparently improve the OS rate of patient with LUAD. The 13 hub genes, namely, *CCT6A*, *CDK5R1*, *CEP55*, *DNAJB4*, *EGLN3*, *HDGF*, *HOXC8*, *LIMD1*, *MKI67*, *PCP4L1*, *PPIL1*, *SCAI*, and *STK32A*, showed a correlation with the OS status.

**Conclusion:**

7 miRNAs were identified as novel biomarkers for the prognosis of patients with LUAD. This study offered a deeper comprehension of LUAD treatment and prognosis from the molecular level and helped enhance the understanding of the pathogenesis and potential molecular events of LUAD.

## 1. Introduction

Lung carcinoma is among the commonest malignancies that exert tremendous social and economic influence upon patients and their families [1]. As a common lung carcinoma form, non-small-cell lung cancer (NSCLC) can be further divided into adenocarcinoma (LUADs) and squamous cell carcinoma (LUSCs). Despite decades of progress in early detection and treatment, the survival rates of patients in advanced stages remain low [2]. Effective biomarkers to identify patients who may have greater possibilities of recurrence and risk of death are also lacking. LUAD is the most aggressive histologic kind of lung carcinoma. The incidence of LUAD is also increasing year by year [3]. Given that early detection and effective treatments are lacking in the early stages of this disease, its mortality rate has not decreased. Therefore, it is imperative to further study the occurrence and development mechanism of LUAD.

MicroRNAs (miRNAs) refer to small noncoding RNAs whose lengths range from 18 nucleotides to 25 nucleotides. They regulate gene expression at the posttranscriptional level by binding to the 3′-untranslated region of target miRNAs, resulting in mRNA degradation, cleavage, or translation inhibition [4]. The silencing complex degrades the mRNA or prevents its translation under the guidance of miRNA by pairing with the mRNA base of the target gene. miRNAs are capable of acting as tumor suppressors or oncogenes by regulating genes involved in tumorigenesis. Abnormal miRNA expression is associated with most cellular functions, especially those related to the occurrence and progression of cancer, thus enabling miRNA to be an attractive biological marker in the detection, classification, and prediction of diverse carcinomas [5–7]. Earlier studies have attempted to identify miRNAs served as potential biomarkers in lung carcinoma patients. Bishop et al. [8] found the usability of methods upon the basis of miRNA for the classification of LUSC and LUAD. Li et al. [9] identified eight miRNA signatures as latent biomarkers to predict the survival status in LUAD patients.

Although there has been considerable progress in the systematic evaluation of carcinoma-related miRNAs and molecular markers to predict overall survival (OS) or immunotherapy response in patients with LUAD, for example, Zhong et al. [10] systematically revealed 38 common regulatory miRNAs in cancer tissues and circulation by integrating literatures. However, more diagnostic and therapeutic miRNA biomarkers are still needed for professional in-depth clinical evaluation to support personalized treatment for lung cancer patients, which needs to be confirmed by randomized multicenter clinical trials.

The Cancer Genome Atlas (TCGA) database possesses massive standardized clinical data, such as gene expression information, miRNA expression data, DNA methylation information, and tremendous samples from every kind of carcinoma [11]. In the present study, RNA sequencing and miRNA sequencing of data from TCGA were used to display the dysfunctional miRNA microenvironment and establish helpful biological markers for treatment with miRNA.

## 2. Methods

### 2.1. Data Collection

Raw data of miRNA and mRNA expression and other clinicopathological information for LUAD was acquired from TCGA data portal. R package was used to isolate LUAD tissues from adjacent nonneoplastic lung tissues in the downloaded sample. Finally, 594 mRNA LUAD-related samples (59 normal vs. 535 tumors) and 567 miRNA LUAD-related samples (46 normal vs. 521 tumors) were downloaded from TCGA online database. The clinicopathological data collected included sex, age, stage, and TMN stage, as shown in Table 1. All data were from TCGA, and they did not require further IRB approval. This study complied with TCGA's publication guidelines and data access policies.

### 2.2. Establishment and Validation of Prognostic Signature Based on miRNA

R-Pack (edgR) was used for the differential analysis of mRNA expression data, and mRNAs possessing apparently distinctive expression levels were selected (FDR < 0.05) and |log2FC| ≥ 1.0. Normalization of the expression profiles was performed for miRNA through the R package. Then, the profiles were classified into two groups: testing group and training group. We used the Cox univariate proportional risk regression to evaluate the miRNA levels, T, N, M, age, sex, staging, and survival status in the training group. Multivariate Cox analysis was utilized for those with *P* < 0.05. Only miRNAs and clinical factors for which *P* < 0.05 in the univariate and multivariate Cox analyses were thought to be prognostic factors for LUAD. The prognostic features were calculated as follows: risk score = (coefficient miRNA1 × expression of miRNA1) + (coefficient miRNA2 × expression of miRNA2) + ⋯+(coefficient miRNA*n* × expression miRNA*n*). We divided patients with LUAD into high-risk and low-risk groups based on the median risk score. Kaplan-Meier analysis was used to analyze and compare overall survival (OS) times between the two subgroups with a two-side log-rank test. Time-dependent receptor operating characteristic (ROC) curves were conducted to assess the specificity and sensitivity of prognostic features based on miRNA expression.

### 2.3. Bioinformatic Analysis of miRNA Target Genes and Pathways

Three online analysis approaches were employed to forecast and ensure the completeness of the target genes: miRDB (http://www.mirdb.org/miRDB/), TargetScanHuman (http://www.targetscan.org/), and miTarBasee (http://mirtarbase.mbc.nctu.edu.tw/). David Database (https://david.ncifcrf.gov/) was used to perform Gene Ontology (GO) analyses, which comprised molecular functionality (MF), biological process (BP), and cell composition (CC), pathways analyzing in the Kyoto Encyclopedia of Genes and Genomes (KEGG). Cytoscape software was employed to visualize the network of interactions in miRNAs and their target mRNAs. The official gene symbols for the predictive target genes were imported into the Search Tool for the Retrieval of Interacting Genes (http://string-db.org) to evaluate the gene interaction status within the protein-protein interaction network.

## 3. Results

### 3.1. Establishing miRNA Prognostic Signature in Association with Survival Status of Patients with LUAD

**Figure 1:**
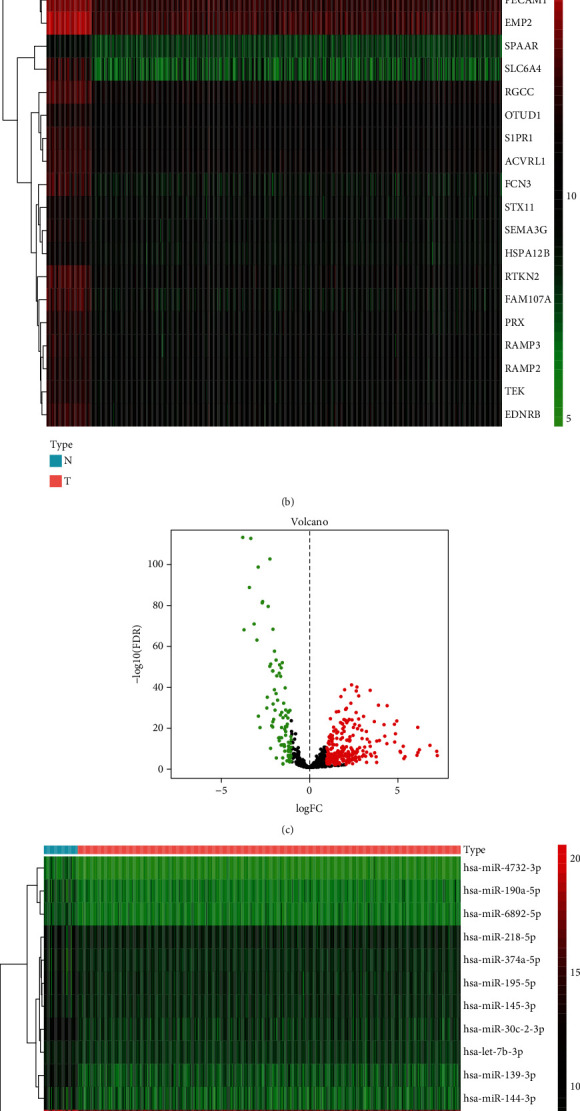
(a) Volcano plot of differentially expressed mRNAs. (b) Heat map of mRNAs with different expressions. (c) Volcano plot of miRNAs with different expressions. (d) Heat map of miRNAs with different expressions. The red color refers to upregulatory mRNAs/miRNA, and the green color means downregulatory mRNAs/miRNA. mRNAs: messenger RNAs; miRNAs: microRNAs.

**Figure 2:**
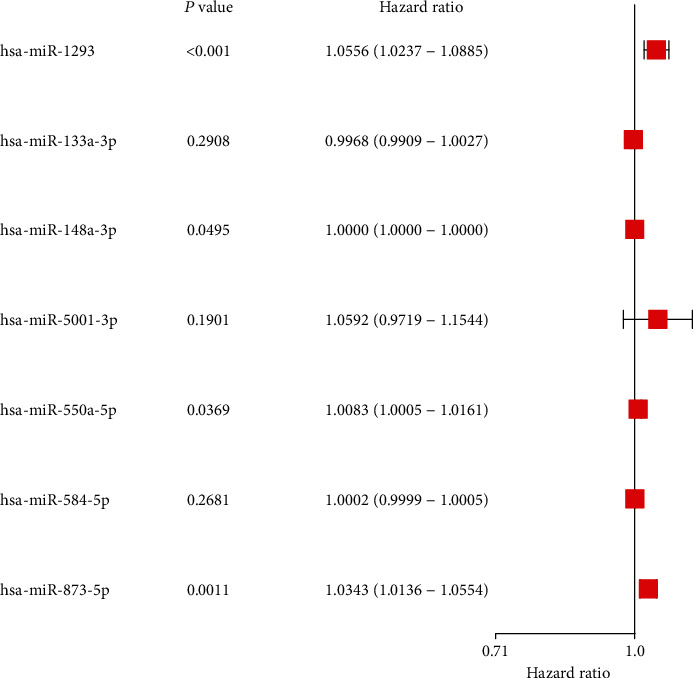
Multivariate Cox analysis to identify differentially expressed miRNAs.

A total of 5523 differentially expressed mRNAs, among which 3711 were upregulated and 1812 were downregulated (Figures 1(a) and 1(b), Table S1), and 362 differently expressed miRNAs (266 upregulated and 96 downregulated miRNAs, Table S2) were acquired (Figures 1(c) and 1(d)). Subsequently, characteristics of the differently expressed miRNAs were identified through univariate Cox analysis (Table S3). Then, important miRNAs from the univariate Cox regression models and clinical factors were used in the multivariate Cox proportional hazard regression models. Seven miRNAs with different expression levels (hsa-miR-1293, hsa-miR-5001-3p, hsa-miR-550-5p, hsa-miR-584-5p, hsa-miR-873-5p, hsa-miR-133a-3p, and hsa-miR-148a-3p) were selected. These miRNAs were used as the model miRNAs (Figure 2). The prognostic features were calculated as follows: risk score = (5.412*e* − 02 × expression of hsa − miR − 1293) + (5.754*e* − 02 × expression of hsa − miR − 5001 − 3p) + (8.253*e* − 02 × expression of hsa − miR − 550 − 5p) + (1.908*e* − 04 × expression of hsa − miR − 584 − 5p) + (3.372*e* − 02 × expression of hsa − miR − 873 − 5p) + (−3.203*e* − 03 × expression of hsa − miR − 133a − 3p) + (−1.337*e* − 06 × expression of hsa − miR − 148a − 3p).

### 3.2. Survival Outcome and Multivariate Examination

**Figure 3:**
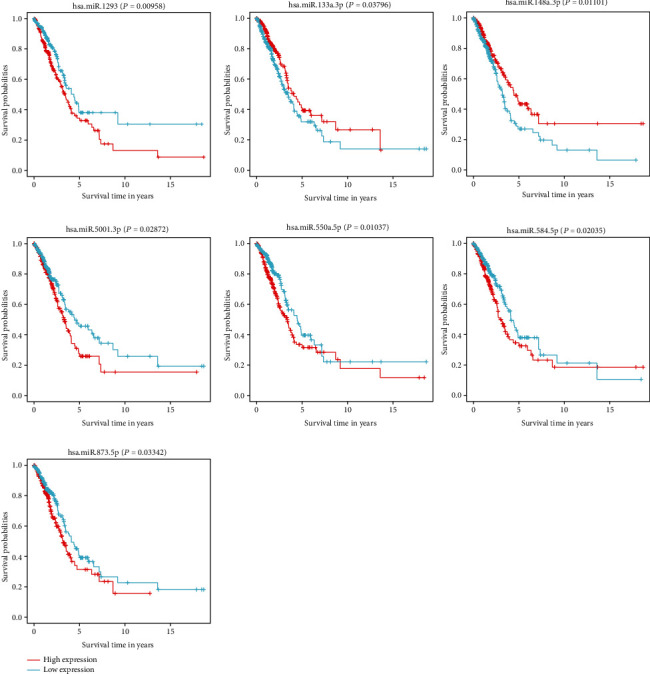
Kaplan-Meier surviving curves of 7 miRNAs composing the prognostic signature of LUAD.

**Figure 4:**
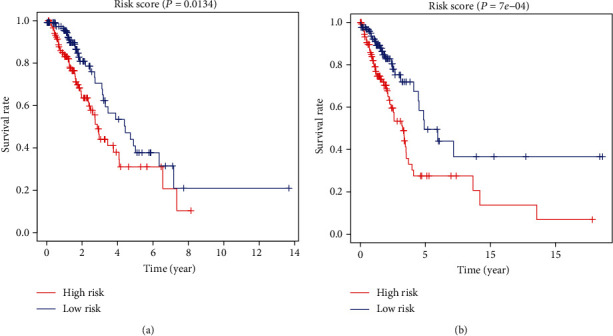
Overall surviving analysis for the training group and testing group: (a) training group and (b) testing group.

**Figure 5:**
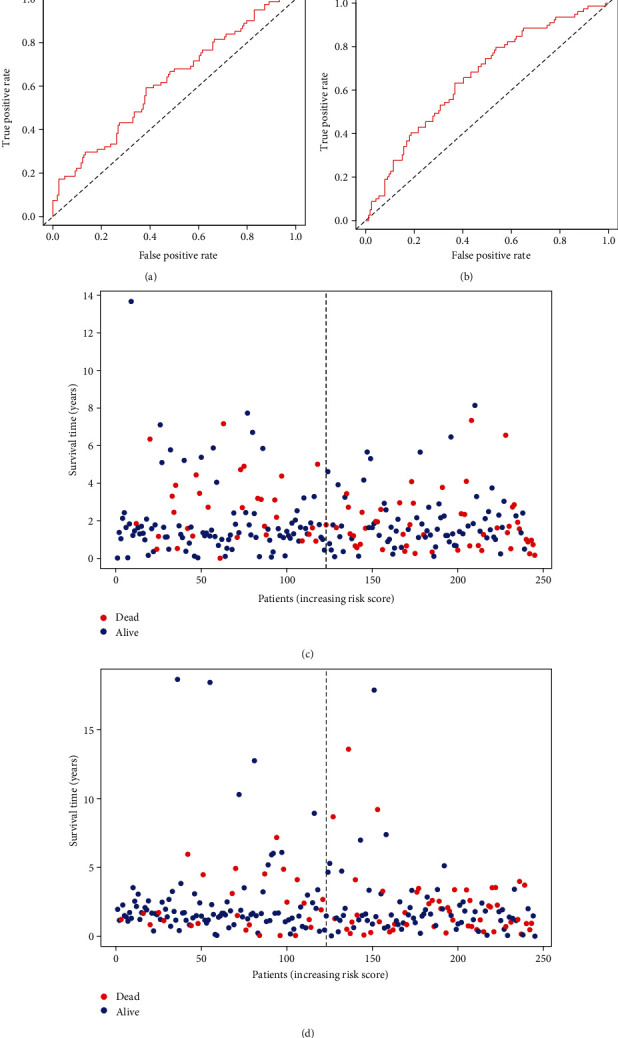
ROC curves and patient's risk surviving status plot in the training group and testing group. (a) ROC in the training group. (b) ROC in the testing group. (c) Surviving status in the training group. (d) Surviving status in the testing group.

**Figure 6:**
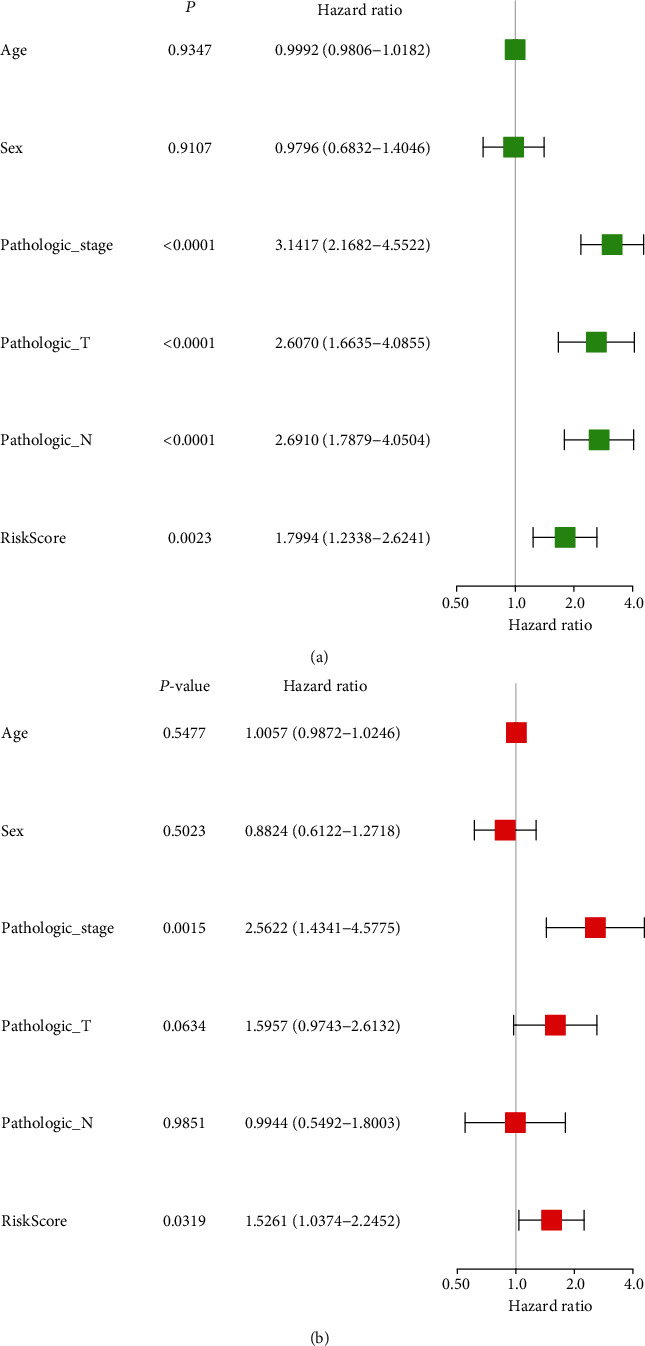
Univariate and multivariate Cox analyses for identifying the risk factors. (a) Univariate. (b) Multivariate.

The effect of the expression of seven miRNAs on the survival of patients was analyzed using the Kaplan-Meier curve. As shown in Figure 3, these seven miRNAs significantly influenced the OS outcomes. Patients with LUAD were divided into low-risk and high-risk subgroups based on the median calculated using the risk score formula. The results showed that the OS of patients in the high-risk group was lower than that of patients in the low-risk group (*P* = 1.34*e* − 02 and *P* = 7*e* − 04, Figure 4). Time-dependent ROC curve analyses were used to evaluate the sensitive and specific features of the 7 miRNA signatures in predicting the prognosis The area under curve (AUC) of ROC was 0.617 in the training group and 0.661 in the testing group at 5-year OS (Figures 5(a) and 5(b)), indicating the moderateness of this prognostic model in terms of sensitivity and specificity. The risk scores of the training and testing group were sorted, and the survival status of each patient was plotted on a heat map. An apparently higher mortality was seen in the high-risk group compared with the low-risk group (Figures 5(c) and 5(d)). Risk factor was identified, and a prognostic model was developed through univariate and multivariate Cox analyses based on these 7 miRNAs. In accordance with the characteristics of the seven miRNAs, risk score (HR = 1.5261, 95%CI = 1.5639–4.0761, and *P* = 0.0319) and pathological stage (HR = 2.5622, 95%CI = 1.4341–4.5775, and *P* = 0.0015) were found to be independent prognosis factors for OS (Figures 6(a) and 6(b)).

### 3.3. GO and KEGG Enrichment Analyses of Target Genes

**Figure 7:**
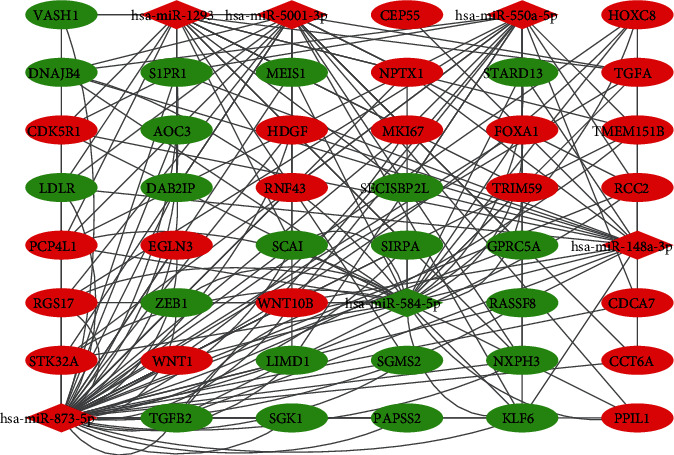
Cytoscape studies the latent link amid microRNA and target genes. Redness color refers to upregulation, and greenness color presents downregulation.

**Figure 8:**
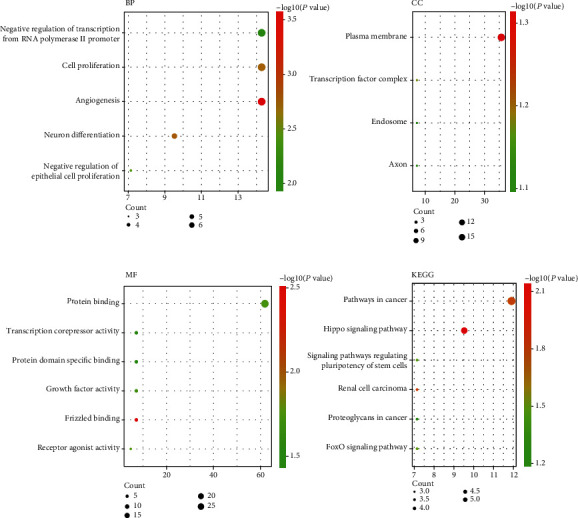
GO and KEGG pathways of target genes under the regulation of the 7 prognosis-related miRNAs.

Three independent websites were used to predict the target genes, and the potential biological functions of the seven miRNAs in the development of LUAD were determined. The overlapping genes were identified as hub genes. A total of 42 genes were revealed as regulated by the 7 miRNAs (Table S4). As shown in Figure 7, hub miRNAs regulated a range of genes, some of which were regulated by two or more miRNAs. Interestingly, miR-873-5p regulated 36 genes, accounting for 85.7% of the prognosis-related miRNAs. Then, these target genes were functionally enriched through GO and KEGG categories. According to Figure 8, the outcomes of the GO analyses demonstrated unbalanced enrichment of genes throughout three biological statuses. Biological process (BP) analyses presented the enrichment of target genes during angiogenesis and cell proliferation and negatively regulated the transcription from RNA polymerase II promoter. Cellular component enrichment showed the main enrichment of genes in plasma membrane, and molecular function analyses demonstrated the main focus of target genes was on protein binding. KEGG enrichment showed that those genes may contribute to LUAD tumorigenesis through multiple pathways related to carcinoma, such as pathways in carcinoma, the Hippo signaling pathway, and the FoxO signaling pathway.

### 3.4. Survival Outcomes of MicroRNA Target Genes

**Figure 9:**
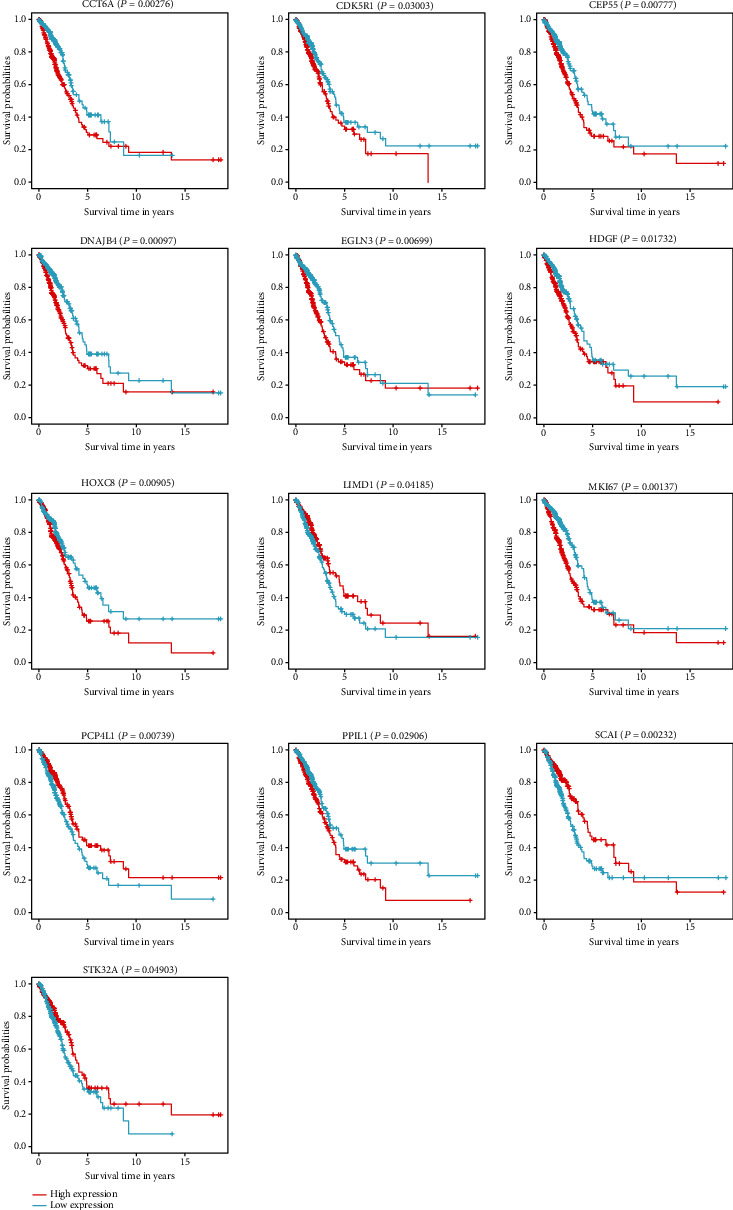
Overall survival analysis of identified target genes and the protein-protein interaction (PPI) network.

According to the analyses of the influence of target gene expression on survival outcomes, the expression of 13 genes, namely, *CCT6A* (*P* = 0.00097), *CDK5R1* (*P* = 0.03003), *CEP55* (*P* = 0.00777), *DNAJB4* (*P* = 0.00097), *EGLN3* (*P* = 0.00699), *HDGF* (*P* = 0.01732), *HOXC8* (*P* = 0.00905), *LIMD1* (*P* = 0.04185), *MKI67* (*P* = 0.00137), *PCP4L1* (*P* = 0.00739), *PPIL1* (*P* = 0.02906), *SCAI* (*P* = 0.00232), and *STK32A* (*P* = 0.04903), played an important role on OS (Figure 9).

## 4. Discussion

As the main regulator of many biological and pathological processes, miRNAs are the focus of research on tumor genesis and development. Diverse evidence suggested that miRNAs establish a complicated combination of gene expression and pathway regulations, prognostic factors, and therapeutic targets in different kinds of carcinomas, such as lung carcinoma. These potential miRNAs can be used for the early detection, molecular classification, prognostic prediction, and therapeutic efficacy of lung cancer [12]. To date, some miRNAs with prognostic value in NSCLC were identified in several studies, including miR-21, miR-200c, miR-125b, miR-148b, miR-365, miR-124, miR-32, and miR-146a [13]. LUAD is characterized by advanced and metastatic tumors that have poor survival outcomes compared with other carcinomas, and the 5-year survival rate is lower than 18% [14]. Thus, understanding the fundamental mechanisms of miRNA regulation could provide a helpful way to develop LUAD therapies possessing great effectiveness. Under the Cox regression model of TCGA data, prognostic characteristics based on miRNA have been found in more and more malignant tumors [15, 16]. It was also confirmed that miR-103a-3p, miR-152, miR-152-3p, miR-15b, miR-16, miR-194, miR-34b, and miR-506 could affect the expression of programmed cell death ligand 1 and programmed cell death receptor [10]. Additionally, high expression of miR-155, miR-17-3p, miR-106a, miR-93, and miR-21 and low expression of let-7a-2, let-7b, and miR-145 are associated with adverse outcomes in LUAD patients [17, 18]. However, few reports studied the prognostic characteristics of miRNAs in LUAD based on TCGA data. The present study established a novel and effective miRNA prognostic signal with good prognostic value. The signature indicated miRNA status in patients with LUAD and provided a potential biomarker for therapeutic interventions.

Through Cox regression analysis and Kaplan-Meier curves, 7 miRNAs that could significantly affect OS results were identified, including hsa-miR-1293, hsa-miR-5001-3p, hsa-miR-550-5p, hsa-miR-584-5p, hsa-miR-873-5p, hsa-miR-133a-3p, and hsa-miR-148a-3p. Some of which have earlier association with the molecular mechanisms of tumors. Previous studies found that miR-1293 inhibited the growth of tumor cells by simultaneously targeting *BRD4*, *APEX1*, *RPA1*, and *POLD4* via inhibiting the DNA repair pathway. Luo et al. [19] found that miR-1293 was capable of working as a prognostic biological marker for papillary renal cell carcinoma. Through bioinformatic method, miR-1293 was found to be highly expressed in renal cell carcinoma, and the survival rate of the group possessing high-level miR-1293 expression was worse versus that of the group possessing low-level miR-1293 expression. These outcomes revealed the upregulation of miRNA-1293 in cancer and the association of high-expression miR-1293 with poor prognosis. Chen et al. [20] found miR-1293 promoted the proliferation, migration, and invasion of LUAD cells via targeting PGM5, and high expression of miR-1293 was positively correlated with pathological stage and overall survival difference in LUAD patients, suggesting that miR-1293 may be an oncogene in the development of LUAD. While studying the role of miR-550a-5p in tumors, the researchers found that overexpression of miR-550a-5p in A549 cells promoted tumor proliferation, while inhibition of miR-550a-5p in H1299 cells inhibited tumor proliferation. miR-550a-5p was proved to promote the development of LUAD through silencing *LIMD1* [21]. This finding is consistent with our present study. Abnormal expression of miR-584-5p recently existed in various human tumors, such as gastric carcinoma, neuroblastoma, medulloblastoma, and lung adenocarcinoma [22–25]. miR-584-5p was found to have a key function in the development of diverse carcinoma through the regulation of distinctive target mRNAs. A decrease in miR-584-5p could be seen in the tumor tissues of patients with NSCLC and cell lines under MMP-14 regulation [26] or YKT6 targeting [27]. In the present study, miR584-5p expression was lower expression compared with that in normal noncancer samples, indicating that miR-584-5p behaves as a tumor suppressor and is a latent molecular biological marker among patients with LUAD. Low miR-133a-3p expression has been found widespread in diverse carcinomas, such as renal cell carcinoma, colorectal cancer, and prostate cancer, and it predicted inferior prognosis [28–30]. However, some evidence demonstrated high miR-133a-3p expression among hepatocellular carcinomas, multiple myeloma, breast carcinoma, and osteosarcoma [31–34], indicating the oncogenic or tumor-suppressive miRNA of miR-133a-3p depending on carcinoma types.

At present, the relationship between hsa-miR-5001-3p and cancer has not been reported, and it still needs in-depth study by researchers. In addition, the miRNAs identified in this study have inconsistencies with the expression in noncancerous tissues. Growing evidence showed that miR-873 has an important function as a tumor suppressor among several human cancers. For example, overexpression of miR-873 could reduce proliferation, migration, and invasion of glioblastoma pleomorphic cells by regulating IGF2BP1 expression [35], such as in colon cancer. However, other studies suggested that miR-873 works as an oncogene. As for hepatocellular carcinoma, upregulated level can be seen in miR-873 expression in tissues and cells, and downregulated miR-873 could inhibit cell growth and metastasis [36]. Gao et al. [37] confirmed that miR-873 could boost the proliferation and migration of LUAD cells, consistent with our results of the present study. Similarly, miR-148a-3p was found lower expression in tumors, and the related expression of miR-148a-3p in esophageal cancer samples was below the level of cancerous tissues [38]. Expression of miR-148a-3p was also reduced in epithelial ovarian carcinoma tissues, and low miR-148a-3p expression had an association with increased OS [39]. However, increasingly expressed miR-148a-3p was found in other carcinoma tissues. Hua et al. [40] revealed that miR-148a was high expression in glioblastoma by regulating the occurrence and development of glioma cells and in osteosarcoma samples [41]. miR-148a-3p has also been studied among NSCLC. Xie et al. [42] reported miR-148a-3p prevented NSCLC from proliferating and epithelial-mesenchymal transition progression through modulating the Ras/MAPK/Erk signaling pathway. In conclusion, the findings of bioinformatic analysis in this study demonstrated that these miRNAs have carcinogenic or anticancer effects in the development of various cancers through different mRNAs.

The target genes of these 7 miRNAs were identified, GO annotation and KEGG enrichment analyses were performed, and to further understand the role and mechanism of these miRNAs in LUAD carcinogenesis, we mapped the interaction network. Annotation analysis conducted on DAVID, and the results showed that the target genes of these miRNAs were involved in significant BPs that may be related to carcinogenesis. KEGG pathway analyses showed the main enrichment of these target genes in carcinoma pathways, the Hippo signaling pathway, and the FOXO signaling pathway. The Hippo signaling pathway serves as a new signaling pathway that has a regulatory function on various biological procedures. A growing number of evidence indicated that this pathway could exert an essential role in LUAD development. A recent lung cancer transcriptome meta-analysis showed that several HIPPO pathway component (NF2, LATS1, PTPN14, YAP1, TAZ, TAOK, and FAT1) genes were found to fuse in lung carcinoma, and they were independent prognosis factors for low lung cancer survival [43]. Gobbi et al. [44] showed that the Hippo pathway could regulate the resistance of lung cancer cells to BET protein inhibitors. Human adenocarcinoma-related gene AGR2 induces bidirectional regulatory protein expression through Hippo pathway coactivator YAP1 [45]. FOXO is a subfamily of the forkhead transcription factor family, which has a significant function in cellular fate determination. This subfamily is also thought to play a key functional role as tumor suppressors in a wide range of cancers. Hydroxychloroquine is a classic antimalarial drug used in preclinical studies and clinical trials to treat cancer. It has been reported that it can inhibit lung tumorigenesis by inducing nuclear translocation of FOXO3a [46].

By analyzing the effect of target gene expression on patient survival, 13 gene expression levels were obtained, including those of *CCT6A*, *CDK5R1*, *CEP55*, *DNAJB4*, *EGLN3*, *HDGF*, *HOXC8*, *LIMD1*, *MKI67*, *PCP4L1*, *PPIL1*, *SCAI*, and *STK32A*. The results showed that target genes had a significant association with the 10-year survival rates of patients with LUAD. However, further explorations are still needed to confirm these observations.

To sum up, nine miRNA signatures were constructed based on TCGA dataset, which are capable of being applied as a prognostic factor for patients with LUAD. However, this study also has some shortcomings. The mechanism of miRNA regulation of tumor biological behavior in LUAD cells needs to be verified experimentally. In addition, multicenter clinical cohorts should be used to validate the practicability of prognostic models.

## 5. Conclusions

In summary, bioinformatic method was used to analyze LUAD-related mRNAs and miRNAs in TCGA database in a systematical manner. 7 miRNAs were found to significantly influence OS outcomes in patients with LUAD. This study deepened the understanding on LUAD treatment and prognosis from the molecular level and helped boost the knowledge on the pathogenesis and latent molecular events of LUAD. These findings contributed to the early diagnosis and prognosis of patients with LUAD and laid a foundation for upcoming clinical explorations. However, the mechanism of action of miRNA and the regulatory network of miRNA-mRNA interactions are peculiarly complicated. This study provided theoretical knowledge and analyses of the clinical data. However, additional scientific studies are required to confirm the observations and investigate their clinical application potential in the improvement of the outlook for patients with LUAD.

## Figures and Tables

**Table 1:** The characteristics of LUAD patients in TCGA.

Variable	Number of samples
Gender	
Male/female	174/172
Age at diagnosis	
≤65/>65	166/180
Stage	
T	300
N	291

LUAD: lung adenocarcinoma; TCGA: The Cancer Genome Atlas; T: tumor; N: node.

## Data Availability

The datasets generated and analyzed during the current study are publicly available from the following online databases: TCGA (https://portal.gdc.cancer.gov/repository).

## Abbreviations

miRNAs: MicroRNAs
NSCLC: Non-small-cell lung cancer
LUAD: Lung adenocarcinoma
TCGA: The Cancer Genome Atlas
OS: Overall survival
RNA-seq: RNA sequencing
ROC: Receiver operating characteristic
GO: Gene Ontology
KEGG: Kyoto Encyclopedia of Genes and Genomes
AUC: Area under the curve
BP: Biological processes
MF: Molecular function
CC: Cell component
HTLV-I: Human T-cell leukemia virus type I
PPI: Protein-protein interaction.

## Supplementary Materials

Supplementary 1 Table S1: differentially expressed mRNAs.

Supplementary 2 Table S2: differentially expressed miRNAs.

Supplementary 3 Table S3: the results of univariate Cox analysis.

Supplementary 4 Table S4: online databases to predicted 42 target genes of these seven miRNAs.
